# Asymmetry of mandibular dentition is associated with dietary specialization in snail-eating snakes

**DOI:** 10.7717/peerj.3011

**Published:** 2017-03-02

**Authors:** Masaki Hoso

**Affiliations:** Hakubi Center for Advanced Research, Kyoto University, Kyoto, Japan; Department of Zoology/Graduate School of Science, Kyoto University, Kyoto, Japan

**Keywords:** Predation, Evolutionary novelty, Left–right asymmetry, Adaptation, Specialization

## Abstract

**Background:**

In vertebrates, the left-and-right pairs of homologous organs are generally present in equal numbers. A remarkable exception is snail-eating snakes in the family Pareidae: almost all the pareid snakes have much more teeth on the right mandible than on the left for functional specialization in feeding on the dextral majority of land snails. Because the only exceptional species with symmetric dentition has been regarded as a slug-eater, the extent of dietary specialization on slugs could shape the degree of the lateral asymmetry of mandibular dentition (dentition asymmetry) even among snail eaters.

**Methods:**

To test this, I compared the morphology and behavior of two sympatric species of Taiwanese snail-eating snakes, *Pareas atayal* and *P. formosensis*.

**Results:**

Specimens collected in the same locality showed that the dentition asymmetry of *P. formosensis* was significantly smaller than that of *P. atayal*. Congruent to its weak asymmetry, *P. formosensis* showed a strong preference of slugs to snails in the feeding experiment.

**Discussion:**

The dietary specialization of *P. formosensis* on slugs would contribute to niche partitioning from the sympatric congener *P. atayal*. This study suggests that the diverse variation in the dentition asymmetry of pareid snakes is the result of their dietary specialization and divergence.

## Introduction

Vertebrates are generally characterized by the lateral symmetry of external morphology; however, diverse bilaterian animals represent conspicuous morphological differentiations in their left-and-right pairs of organs (Palmer, 1996; Palmer, 2005). The head parts of flatfish and the tusk of the narwhal are striking examples. However, even in these cases, asymmetric components of the paired organs are present in equal numbers since the early stage of their development. A notable exception is the tooth number of snail-eating snakes in the family Pareidae (often misdesignated as Pareatidae (Savage, 2015) in SE Asia, showing substantial difference between the left and right mandibles (dentition asymmetry: DA) (Hoso, Asami & Hori, 2007).

Pareid snakes have been widely regarded as dietary specialists of land snails and slugs (Pough et al., 2016). Their DA has been considered feeding specialization on the dextral majority of land snails (Hoso, Asami & Hori, 2007). They usually attack the soft body of a crawling snail and extract it by protracting and retracting their mandibles through the aperture (mouth-opening) of the shell (Götz, 2002; Hoso, Asami & Hori, 2007). Almost all pareid species have a greater number of mandibular teeth on the right side compared with the left, and the only exception is a slug-eating specialist *Asthenodipsas malaccanus* in the Malay Peninsula and the Greater Sunda Islands (Stuebing & Inger, 1999) that shows completely symmetric dentition (Hoso, Asami & Hori, 2007). Thus, the inter-specific variation in the extent of snail-eaters’ DA might reflect their specific feeding habits or, in particular, the proportion of slugs in their prey contents (Hoso, Asami & Hori, 2007; Hoso et al., 2010).

Here I tested the above hypothesis using two pareid snakes, *Pareas atayal* and *P. formosensis,* that co-occur in the northern part of Taiwan Island. These congeneric species have been recently assigned into phylogenetically distinct, non-sibling species (You, Poyarkov & Lin, 2015). The morphology of museum-preserved specimens collected at the same locality were compared. Live, wild-caught animals were used to investigate the potential difference of their prey preference (snails or slugs) in captivity.

## Materials & Methods

### Snake species

*P. atayal* and *P. formosensis* are indigenous to Taiwan Island, but *P. atayal* only occurs in its northern part. *P. atayal* is the sibling species of *P. iwasakii* for which there is much morphological, behavioral and ecological information (Hoso & Hori, 2006; Hoso, 2007; Hoso, Asami & Hori, 2007; Hoso et al., 2010). Although I mainly focused on the difference in the DA of these two species in this study, differences in other morphological features related with feeding habitats might be identified in further analyses.

### Specimen examination

A total of 51 ethanol-preserved specimens, comprising 38 *P. atayal* and 13 *P. formosensis* were used in specimen examination. They were collected from a small geographic range along a mountain road of approximately 16 km long (7.0 km in distance) in the northern Taiwan Island and preserved in three museums (Kyoto University; National Museum of Natural Science, Taiwan; and National Taiwan Normal University). Snout-ventral length (SVL) was measured and used as an indicator of the body size. Mandibular tooth numbers were counted using a 3-D imaging software (Molcer Plus v.1.35; White Rabbit Co., Ltd., Japan) from CT images of specimens scanned by a µCT scanner (ScanXmateA080S; Comscantecno Co., Ltd., Japan; housed at Kyoto University). An asymmetry index was used to illustrate the extent of DA, represented by 100 magnitudes of the subtraction of left-tooth number from right-tooth number divided by their sum.

Snakes, excepting venomous snakes with fangs, generally possess nearly homodont teeth on their mandibles. Because pareid snakes are non-venomous and seemingly exhibit no specialized teeth, all the teeth examined in this study are considered as serial homologues. Because replacements of teeth occur permanently in snakes, the count of teeth number is unlikely affected by age or conditions of the snake specimens.

Although not adequately documented, Hoso, Asami & Hori (2007) showed considerable intra-specific variations in each species by standard errors. They likely resulted from the geographic variation within species, because the specimens examined in that study were collected across the distribution ranges of pareid snakes. Thus, only snakes collected in the same locality were used for the strict comparisons in this study.

### Feeding experiment

The feeding experiment was conducted between May and September 2016 in a room maintained at 25 °C with L12: D12 circadian rhyme at Kyoto University. A total of 24 living snakes collected from the northern Taiwan, comprising 16 *P. atayal* (194–465 mm in SVL) and 8 *P. formosensis* (223–425 mm), were used. Wild-captured snakes were suitable to test prey preference, because neither its genetic nor epigenetic determinants mattered in this study. Snakes were housed individually in terraria (230 mm width × 153 mm length × 173 mm height). Either three snails or three slugs were randomly selected by size and placed in each terraria just after the snake shed its skin, and whether the snake consumed at least one prey item in three days was recorded. This period was ideal to strictly control the food demands of snakes. Their starvation condition was expected to be severe and similar because snakes generally eat nothing for approximately ten days before skin shedding. Every snake experienced both treatments of three snails and three slugs randomly selected by size.

Snails of *Acusta despecta* (8.0–20.0 mm in shell diameter) collected on Okinawa Island, Japan, and a slug *Meghimatium bilineatum* (0.3–1.2 g in wet weight) collected in Kyoto, Japan were used as prey animals. Because both species distribute sympatrically with the *Pareas* snakes (Hsieh, Wu & Tsai, 2013) and are abundant in rural regions, they can be assumed to be encountered commonly by Taiwanese *Pareas* snakes in the field. Moreover, because I have regularly fed *Ac. despecta* to many species of pareid snakes (*Aplopeltura boa*, *P. boulengeri*, *P. carinatus*, *P. iwasakii*, *P. komaii*, *P. margaritophorus* and *P. stanleyi*) for months to years (M Hoso, pers. obs., 2008–2010), this snail species was considered suitable to test prey preference.

I did not count teeth of snakes used in the behavioral experiment due to a technical difficulty. Their tooth numbers unlikely differed from those of the examined specimens because they belonged to the same populations of each species.

This research adhered to the legal requirements of Japan and Taiwan and was approved by the standardized protocol license No. 104032 of National Taiwan Normal University and the Kyoto University guideline under protocol H2814.

### Statistics

#### For morphological data

First, the null hypotheses of correlations of SVL with left- and right-tooth numbers and asymmetry index of DA in *P. atayal* and *P. formosensis* to confirm negligible ontogenetic changes in tooth number and DA as shown in *P. iwasakii* (Hoso, Asami & Hori, 2007), using Pearson’s correlation coefficient (*r*) and the Kendall rank correlation coefficient (*τ*) for tooth numbers and asymmetry index, respectively. The DA of each species was tested using a Welch 2-sample *t*-test. Third, the inter-specific difference of DA, in terms of asymmetry index, was examined. Generalized linear models (GLMs) were used to incorporate species or none as a variate, and the sum of the left- and right-tooth numbers as an offset to account for the subtraction of left-tooth number from right-tooth number. The Poisson distribution was applied as the probability distribution to all GLMs. A likelihood-ratio test was performed between the full model and the test model where species was not incorporated as a variate. Finally, the inter-specific difference of DA was used to compare responsibilities of the left- and right-tooth numbers to DA. Because comparison of coefficients by discriminant analysis was not applicable due to the heterogeneity of variance, the deviance residuals analyzed in GLMs were compared instead. GLMs, as full models, incorporating left- or right-tooth number and species as covariates and the sum of the left- and right-tooth numbers as an offset to account for the subtraction of left-tooth number from right-tooth number. Then, deviance residuals of the models where species were not incorporated were compared and the Poisson distribution was applied as probability distribution to all GLMs.

#### For behavioral data

Due to the zero-inflation, feeding experiment data was analyzed by performing a Bayesian inference on a generalized linear mixed model (GLMM) for 100,000 iterations with a long burn-in of 50,000 and a thinning interval of 50. Snake species, prey species and their interaction were used as fixed effects and snake individuals were a random effect. Binomial distribution was applied as the probability distribution to the GLMM. All the data were analyzed using R v. 3.3.1 (R Development Core Team, 2016) for WINDOWS. The behavioral data were analyzed using the MCMCglmm package for R.

## Results

**Figure 1:**
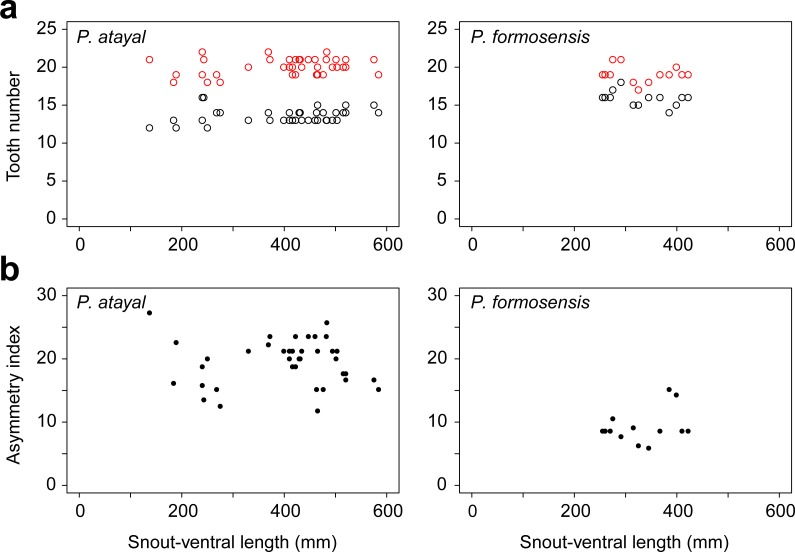
Tooth number and dentition asymmetry of the snakes in association with snout-ventral length (SVL). (A) The number of teeth on the right (red) and left (black) mandibles. (B) The extent of asymmetry (as denoted by the asymmetry index) of *P. atayal* and *P. formosanus*.

**Figure 2:**
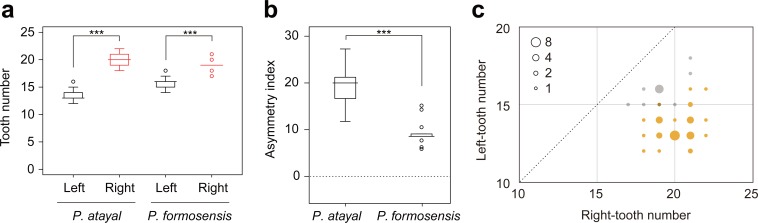
Comparison of mandibular tooth numbers and its DA between *P. atayal* and *P. formosanus*. (A, B) Box plots summarizing the distributions of (A) the tooth numbers and (B) the dentition asymmetry. ^∗∗∗^; *p* < 0.01. (C) 2-D plots of left- and right-tooth numbers of each specimen. The size of circles reflects the sample size, as indicated by the inset legend. Yellow and grey circles correspond to *P. atayal* and *P. formosanus*, respectively. The circle with 15 left and 19 right teeth is only overlapped by each one specimen of the 2 species. The dotted line indicates the symmetry.

There were neither significant correlations of SVL with the numbers of left or right teeth (Fig. 1A; *r* = 0.23, *p* = 0.17 and *r* =  − 0.14, *p* = 0.65 for the right; *r* = 0.20, *p* = 0.22 and *r* =  − 0.41, *p* = 0.17 for the left in *P. atayal* and *P. formosensis*, respectively), nor those with asymmetry index (Fig. 1B; *τ* =  − 0.07, *p* = 0.58 and *τ* = 0.07, *p* = 0.75 in *P. atayal* and *P. formosensis*, respectively) in both snake species. Therefore, I did not considered the effects of body size on tooth number and dentition asymmetry in subsequent analyses. *P. atayal* had 13.6 ± 1.0 left and 20.1 ± 1.1 right teeth (average ± SD) and *P. formosensis* had 15.8 ± 1.0 left and 19.1 ± 1.1 right teeth (Fig. 2A): right-tooth number was significantly greater than left-tooth number in both snake species (Welch 2-sample *t*-test, *p* < 0.01 and *p* < 0.01, respectively). The asymmetry index was significantly smaller in *P. formosensis* (8.6 at the median) than in *P. atayal* (20.0) (Fig. 2B; likelihood-ratio test, *p* < 0.01). The contributions of the left- and right-tooth numbers to DA were separately evaluated by the deviance residuals of each test model. The deviance residual was smaller for left-tooth number than for right-tooth number (Table 1; Fig. 2C), indicating a stronger collinearity of species difference with left-tooth number than with right-tooth number in the models. Raw data are available in Data S1.

**Table 1:** Residual deviance of GLMs accounting for the DA and deviance residuals. See also Fig. 2C.

Fixed effects in each model	Residual deviance	Deviance residuals
Left teeth + Snake species	183.86	–
Left teeth	218.38	34.52
Right teeth + Snake species	878.57	–
Right teeth	340.44	538.13

Analysis of data from the feeding experiment showed that the posterior distribution of the interaction parameter between snake species and prey species deviated significantly from zero (Table 2), demonstrating that prey preferences of *P. atayal* and *P. formosensis* differ from each other. Raw data are available in Data S2.

**Table 2:** The statistical summary of Bayesian inference on a GLMM accounting for prey consumption in the feeding experiment.

Parameters	Posterior distributions			Effective sample size	pMCMC
	Mean	Lower 95% CI	Upper 95% CI		
Snake species	−36.61	−480.50	520.14	24.278	0.840
Prey species	−288.84	−669.28	−13.25	29.167	0.072
Snake × Prey	−4985.69	−7320.74	−2574.67	5.534	<0.001

## Discussion

*P. atayal* exhibited substantial DA with 13.6 left- and 20.1 right-teeth on average, whereas *P. formosensis* had weak DA with 15.8 and 19.1. These tooth numbers are congruent to but slightly larger than those reported by You, Poyarkov & Lin (2015), because they counted only visible teeth (“functional teeth”) from single 3-D model files. The present study counted all teeth in the CT images by optimizing thresholds for voxel visualization. The ranges of DA of *P. atayal* and *P. formosensis* are near the lowest and the highest, respectively, among the genus *Pareas* (Hoso, Asami & Hori, 2007).

The result of the experiment showed a complete avoidance of *P. formosensis* to the snail *Ac. despecta*. Because *Ac. despecta* is a potential natural prey for *P. formosensis* and has been observed to be consumed by many other species of pareid snakes, the present results can generally be assumed to represent the preference of slugs in *P. formosensis*.

The weak DA found in *P. formosensis* is most likely associated with its preference for slugs. To extract the soft body of a snail, pareid snakes alternately protract and retract their mandibles through the aperture of the shell (Hoso, Asami & Hori, 2007). When the shell is clockwisely coiling, the left mandible can be inserted more deeply and move more widely than the right. Thus, the left and right mandibles have been considered to work differently but in concert to improve the intake efficiency. Although kinematic mechanisms have not investigated yet, fewer teeth on the left mandible may be useful to smoothly bite on and off the sticky tissue of a snail and more teeth on the right to grasp it firmly. Therefore, highly asymmetric dentition is undoubtedly irrelevant to consuming slugs, as indicated by the slug-eating pareid snake *As. malaccanus* with symmetric dentition. This significant difference in DA from the co-existing congener *P. atayal* could have resulted in the ecological niche differentiation of these two co-existing snake species by partitioning prey animals.

This study gives rise to a question: if *P. formosensis* is a slug-eating specialist, why does its DA still remain valid? Several hypotheses can be proposed to explain this. First, the weak DA is functionally equivalent to symmetric dentition for slug-eating. Second, the weak DA remains as a result of phylogenetic inertia under relaxed selection on the evolutionary trajectory from its snail-eating ancestor. Third, *P. formosensis* commonly feeds on native snails other than *Ac. despecta*, as well as slugs, in the wild and the weak DA is fine-tuned for that. Even in this case, the present result of the feeding experiment suggests that *P. formosensis* would more often consume slugs. Clearly, more data on the life history including dietary information and prey preference are required to disentangle this enigma of the weak DA in *P. formosensis*.

Dietary information of pareid snakes in the wild is rare in the available literature. The only example of prey identification at the species level is for *P. iwasakii* in Japan. Its prey was identified as a snail (*Satsuma caliginosa*) by an electronic microscopy observation of a radula (and optical observation of a mandible) of a snail collected from feces emitted by a wild-captured snake, and by comparison with reference specimens (Hoso & Hori, 2006). Recently developed high-throughput sequencing techniques, with the aid of a reference library of DNA barcodes, would allow us to identify prey animals from feces (e.g., Shehzad et al., 2012; Boyer et al., 2013; Vesterinen et al., 2013). However, the number of consumed prey of the same species is unlikely to be countable without direct observation of the remnants in the feces (Hoso & Hori, 2006). Using these methods, the dietary information of *P. formosensis* and other pareid snakes could advance our understanding of these species. More specifically, slug-eating of *As. malaccanus* should be quantitatively confirmed in peer-reviewed publications.

Prey preference of pareid snakes was first tested in an experiment using a single individual of *P. iwasakii* and all the 13 species of native snails with over 10 mm of maximum shell diameter (Hoso & Hori, 2006). Of the 13, the snake fed on nine species of the Pulmonata including *Ac. tourannensis*, a congener of *Ac. despecta* used in this study, and *S. caliginosa* that was found in the feces of a wild-captured snake as mentioned above. The other case is described for *P. carinatus* in Thailand. Danaisawadi et al. (2016) indirectly showed that *P. carinatus* avoided *Dyakia salangana* more often than the other five species of potential natural prey (Danaisawadi et al., 2016), although the statistical significance and the number of snakes that attempted to feed on *D. salangana* are uncertain due to the report’s questionable statistics and limited description of methods. The authors asserted that the proximal mechanism of the prey avoidance was the snakes’ visual recognition of *D. salangana* based on its sinistral coiling; however, the report lacked direct tests. Nonetheless, well-designed behavioral experiments for exploring prey preference and its sensory basis will be beneficial in ecological studies of predators in general, especially when their habits are difficult to investigate. Together with the dietary information, more data on prey preference of pareid snakes can help to reveal the association of DA with their dietary specializations and divergence.

Evolution of tooth-number variation is of particular interest in the biology of pareid snakes. Although the present results indicated that the difference of DA between *P. atayal* and *P. formosensis* was primarily due to the divergence of the left-tooth number, this can simply indicate that neither *P. formosensis* increased nor *P. atayal* decreased the left-tooth number from their common ancestor because they are not sibling species (You, Poyarkov & Lin, 2015). Phylogeny-based inference of ancestral states is necessary to reconstruct the trajectory of dentition evolution. Recently, a large-scale, species-level phylogeny of extant snakes (Figueroa et al., 2016) showed a derived phylogenetic position of *As. malaccanus* in Pareidae, implying the secondary evolution of a symmetric dentition from the ancestral DA, as considered previously (Hoso, Asami & Hori, 2007). Likewise, mapping the food habits and left- and right-tooth numbers of each pareid species (considering intra-specific variations) on the tips of the phylogenetic tree will allow us to reconstruct the evolutionary transactions of DA in association with their diversification as molluscivorous specialists in Southeast Asia.

## Conclusions

Congruently to the weak dentition asymmetry, *P. formosensis* showed a strong preference of slugs to snails, leading to niche partitioning from the co-existing snail-eater, *P. atayal*. This study suggests that the inter-specific variation in the dentition asymmetry of pareid snakes has been brought by dietary specialization of each species and dietary divergence between species.

##  Supplemental Information

10.7717/peerj.3011/supp-1 Data S1 Measurements of snake specimens

10.7717/peerj.3011/supp-2 Data S2 Results of the feeding experiment

## Additional Information and Declarations

### Data Availability

The following information was supplied regarding data availability: The raw data has been supplied as Supplementary Files.
